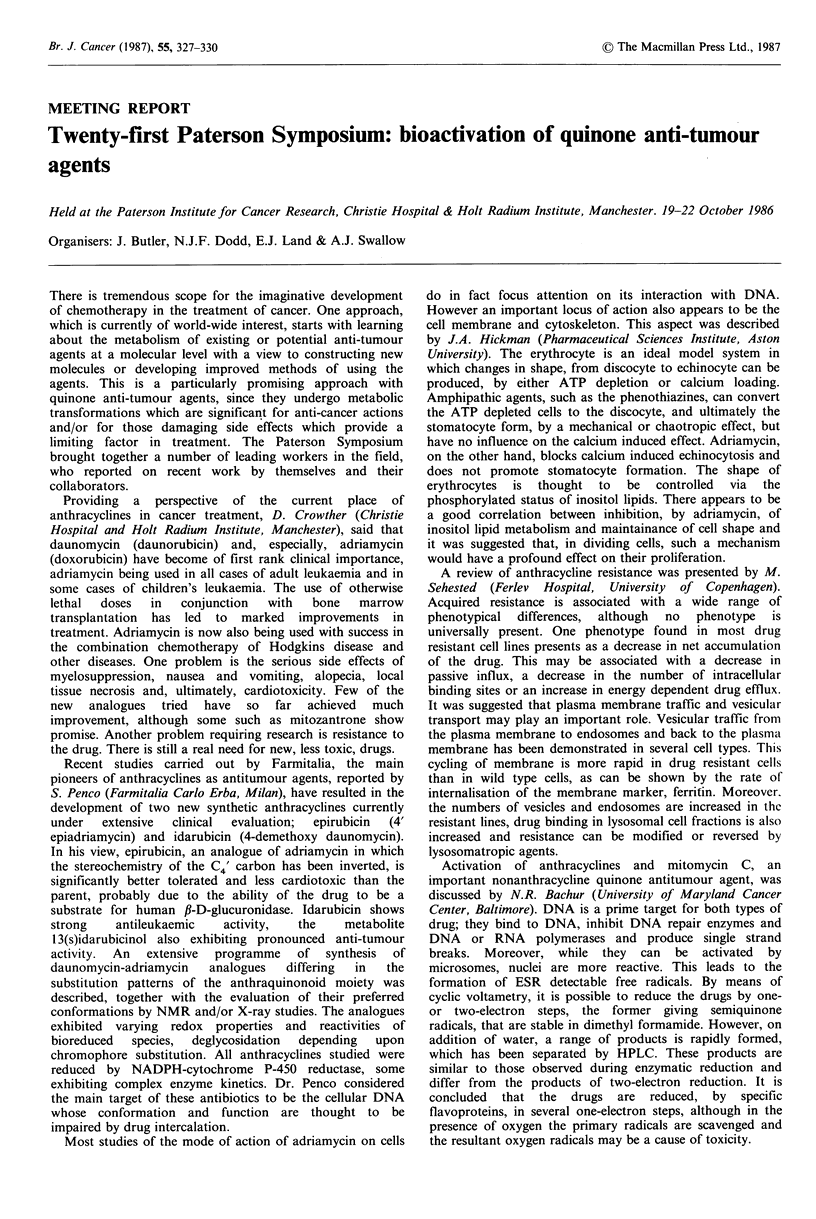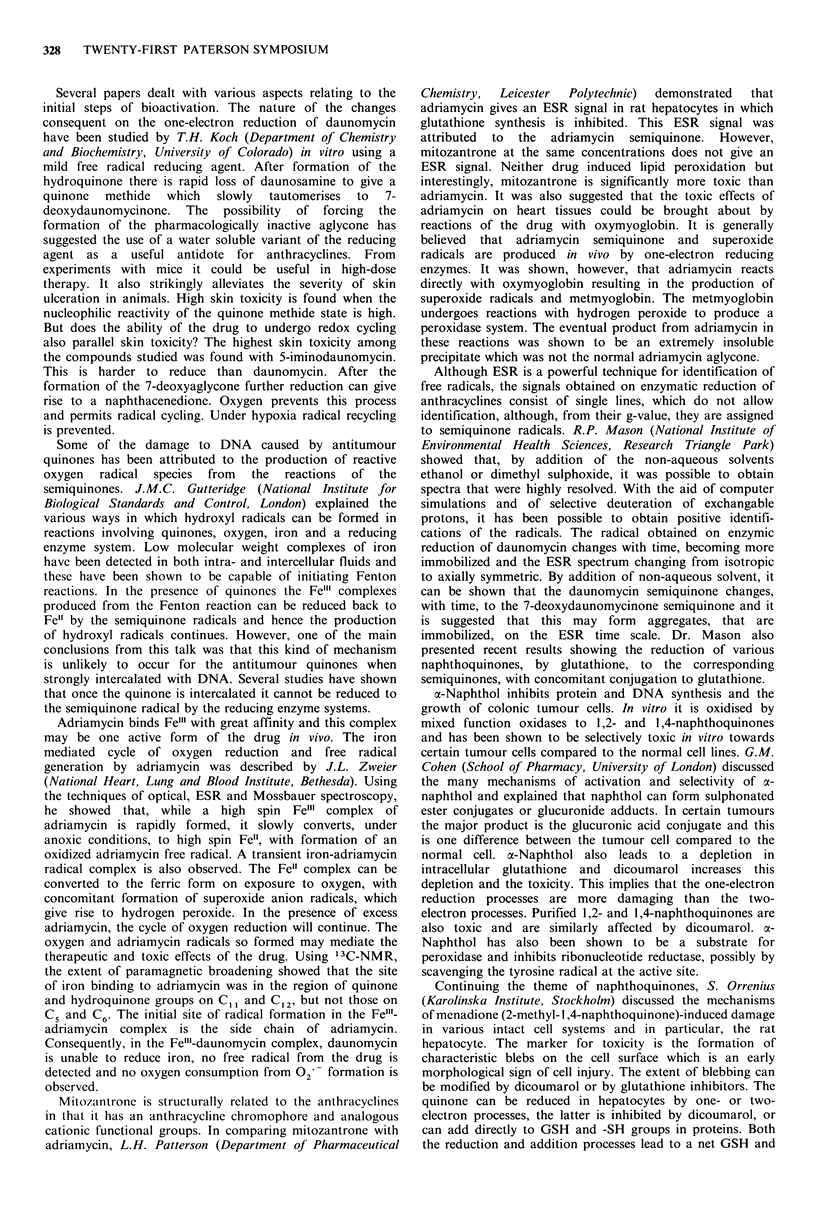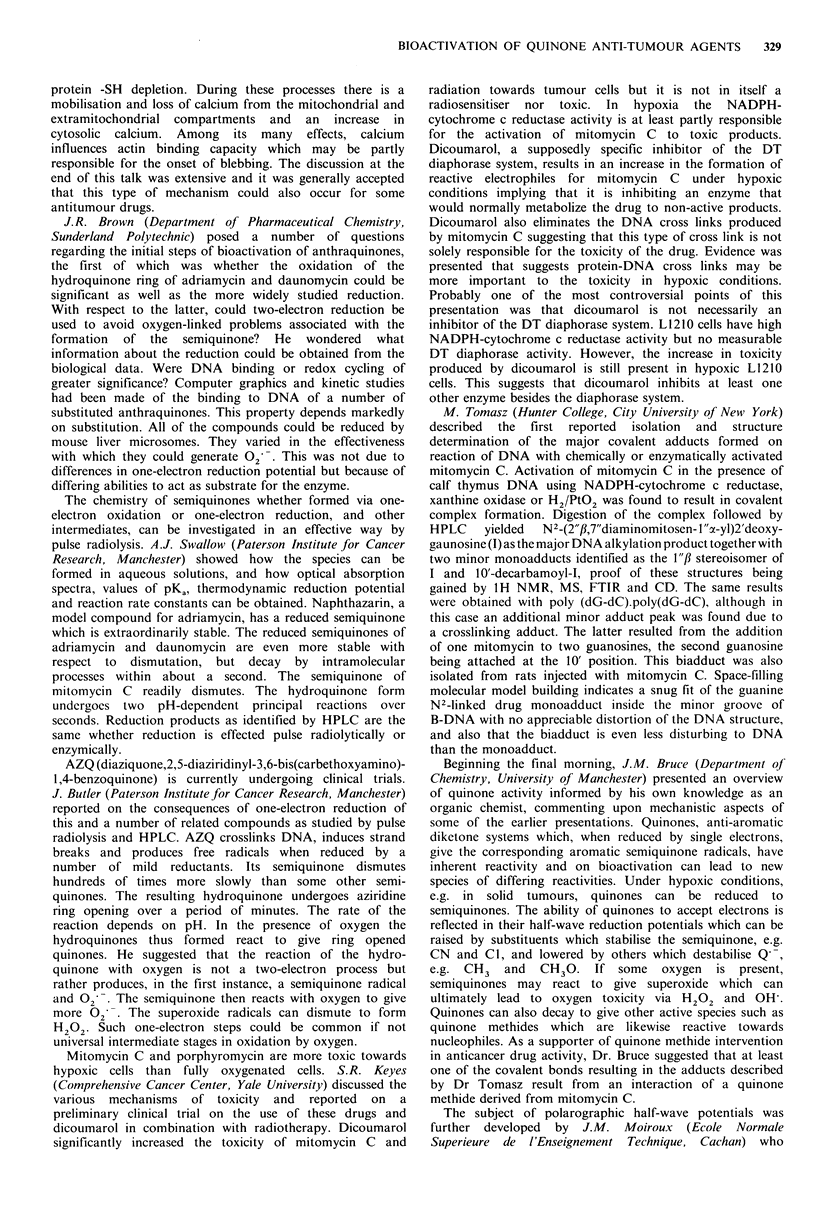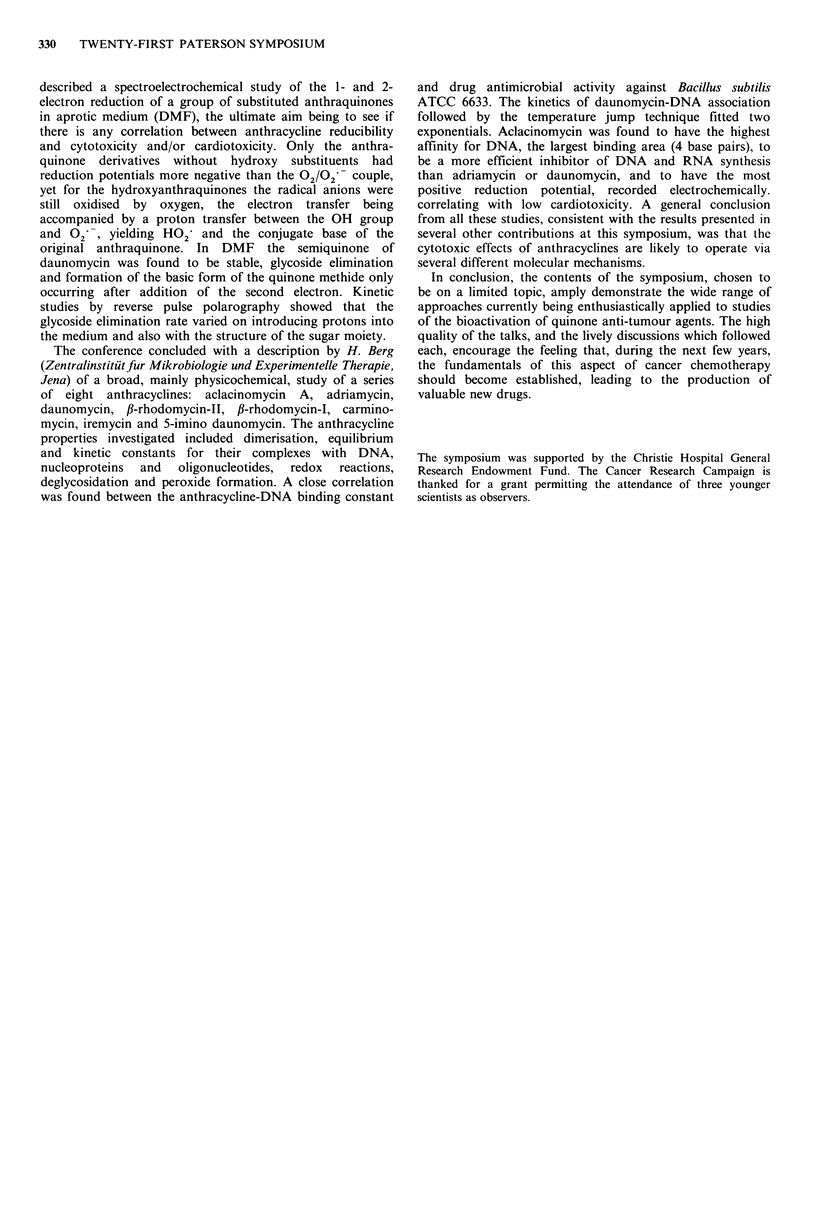# Twenty-first Paterson symposium: bioactivation of quinone anti-tumor agents.

**DOI:** 10.1038/bjc.1987.64

**Published:** 1987-03

**Authors:** 


Br. J. Cancer (1987), 55, 327–330 © The Macmillan Press Ltd., 1987

MEETING REPORT

### Twenty-first Paterson Symposium: bioactivation of quinone anti-tumour agents

*Held at the Paterson Institute for Cancer Research, Christie Hospital & Holt Radium Institute, Manchester. 19–22 October 1986*

Organisers: J. Butler, N.J.F. Dodd, E.J. Land & A.J. Swallow

There is tremendous scope for the imaginative development of chemotherapy in the treatment of cancer. One approach, which is currently of world-wide interest, starts with learning about the metabolism of existing or potential anti-tumour agents at a molecular level with a view to constructing new molecules or developing improved methods of using the agents. This is a particularly promising approach with quinone anti-tumour agents, since they undergo metabolic transformations which are significant for anti-cancer actions and/or for those damaging side effects which provide a limiting factor in treatment. The Paterson Symposium brought together a number of leading workers in the field, who reported on recent work by themselves and their collaborators.

Providing a perspective of the current place of anthracyclines in cancer treatment, *D. Crowther* (*Christie Hospital and Holt Radium Institute, Manchester*), said that daunomycin (daunorubicin) and, especially, adriamycin (doxorubicin) have become of first rank clinical importance, adriamycin being used in all cases of adult leukaemia and in some cases of children's leukaemia. The use of otherwise lethal doses in conjunction with bone marrow transplantation has led to marked improvements in treatment. Adriamycin is now also being used with success in the combination chemotherapy of Hodgkins disease and other diseases. One problem is the serious side effects of myelosuppression, nausea and vomiting, alopecia, local tissue necrosis and, ultimately, cardiotoxicity. Few of the new analogues tried have so far achieved much improvement, although some such as mitozantrone show promise. Another problem requiring research is resistance to the drug. There is still a real need for new, less toxic, drugs.

Recent studies carried out by Farmitalia, the main pioneers of anthracyclines as antitumour agents, reported by *S. Penco* (*Farmitalia Carlo Erba, Milan*), have resulted in the development of two new synthetic anthracyclines currently under extensive clinical evaluation; epirubicin (4′ epiadriamycin) and idarubicin (4-demethoxy daunomycin). In his view, epirubicin, an analogue of adriamycin in which the stereochemistry of the $C_4'$ carbon has been inverted, is significantly better tolerated and less cardiotoxic than the parent, probably due to the ability of the drug to be a substrate for human $\beta$-D-glucuronidase. Idarubicin shows strong antileukaemic activity, the metabolite 13(s)idarubicinol also exhibiting pronounced anti-tumour activity. An extensive programme of synthesis of daunomycin-adriamycin analogues differing in the substitution patterns of the anthraquinonoid moiety was described, together with the evaluation of their preferred conformations by NMR and/or X-ray studies. The analogues exhibited varying redox properties and reactivities of bioreduced species, deglycosidation depending upon chromophore substitution. All anthracyclines studied were reduced by NADPH-cytochrome P-450 reductase, some exhibiting complex enzyme kinetics. Dr. Penco considered the main target of these antibiotics to be the cellular DNA whose conformation and function are thought to be impaired by drug intercalation.

Most studies of the mode of action of adriamycin on cells do in fact focus attention on its interaction with DNA. However an important locus of action also appears to be the cell membrane and cytoskeleton. This aspect was described by *J.A. Hickman* (*Pharmaceutical Sciences Institute, Aston University*). The erythrocyte is an ideal model system in which changes in shape, from discocyte to echinocyte can be produced, by either ATP depletion or calcium loading. Amphipathic agents, such as the phenothiazines, can convert the ATP depleted cells to the discocyte, and ultimately the stomatocyte form, by a mechanical or chaotropic effect, but have no influence on the calcium induced effect. Adriamycin, on the other hand, blocks calcium induced echinocytosis and does not promote stomatocyte formation. The shape of erythrocytes is thought to be controlled via the phosphorylated status of inositol lipids. There appears to be a good correlation between inhibition, by adriamycin, of inositol lipid metabolism and maintainance of cell shape and it was suggested that, in dividing cells, such a mechanism would have a profound effect on their proliferation.

A review of anthracycline resistance was presented by *M. Sehested* (*Ferlev Hospital, University of Copenhagen*). Acquired resistance is associated with a wide range of phenotypical differences, although no phenotype is universally present. One phenotype found in most drug resistant cell lines presents as a decrease in net accumulation of the drug. This may be associated with a decrease in passive influx, a decrease in the number of intracellular binding sites or an increase in energy dependent drug efflux. It was suggested that plasma membrane traffic and vesicular transport may play an important role. Vesicular traffic from the plasma membrane to endosomes and back to the plasma membrane has been demonstrated in several cell types. This cycling of membrane is more rapid in drug resistant cells than in wild type cells, as can be shown by the rate of internalisation of the membrane marker, ferritin. Moreover, the numbers of vesicles and endosomes are increased in the resistant lines, drug binding in lysosomal cell fractions is also increased and resistance can be modified or reversed by lysosomatropic agents.

Activation of anthracyclines and mitomycin C, an important nonanthracycline quinone antitumour agent, was discussed by *N.R. Bachur* (*University of Maryland Cancer Center, Baltimore*). DNA is a prime target for both types of drug; they bind to DNA, inhibit DNA repair enzymes and DNA or RNA polymerases and produce single strand breaks. Moreover, while they can be activated by microsomes, nuclei are more reactive. This leads to the formation of ESR detectable free radicals. By means of cyclic voltametry, it is possible to reduce the drugs by one- or two-electron steps, the former giving semiquinone radicals, that are stable in dimethyl formamide. However, on addition of water, a range of products is rapidly formed, which has been separated by HPLC. These products are similar to those observed during enzymatic reduction and differ from the products of two-electron reduction. It is concluded that the drugs are reduced, by specific flavoproteins, in several one-electron steps, although in the presence of oxygen the primary radicals are scavenged and the resultant oxygen radicals may be a cause of toxicity.

Several papers dealt with various aspects relating to the initial steps of bioactivation. The nature of the changes consequent on the one-electron reduction of daunomycin have been studied by *T.H. Koch* (*Department of Chemistry and Biochemistry, University of Colorado*) *in vitro* using a mild free radical reducing agent. After formation of the hydroquinone there is rapid loss of daunosamine to give a quinone methide which slowly tautomerises to 7-deoxydaunomycinone. The possibility of forcing the formation of the pharmacologically inactive aglycone has suggested the use of a water soluble variant of the reducing agent as a useful antidote for anthracyclines. From experiments with mice it could be useful in high-dose therapy. It also strikingly alleviates the severity of skin ulceration in animals. High skin toxicity is found when the nucleophilic reactivity of the quinone methide state is high. But does the ability of the drug to undergo redox cycling also parallel skin toxicity? The highest skin toxicity among the compounds studied was found with 5-iminodaunomycin. This is harder to reduce than daunomycin. After the formation of the 7-deoxyaglycone further reduction can give rise to a naphthacenedione. Oxygen prevents this process and permits radical cycling. Under hypoxia radical recycling is prevented.

Some of the damage to DNA caused by antitumour quinones has been attributed to the production of reactive oxygen radical species from the reactions of the semiquinones. *J.M.C. Gutteridge* (*National Institute for Biological Standards and Control, London*) explained the various ways in which hydroxyl radicals can be formed in reactions involving quinones, oxygen, iron and a reducing enzyme system. Low molecular weight complexes of iron have been detected in both intra- and intercellular fluids and these have been shown to be capable of initiating Fenton reactions. In the presence of quinones the $Fe^{III}$ complexes produced from the Fenton reaction can be reduced back to $Fe^{II}$ by the semiquinone radicals and hence the production of hydroxyl radicals continues. However, one of the main conclusions from this talk was that this kind of mechanism is unlikely to occur for the antitumour quinones when strongly intercalated with DNA. Several studies have shown that once the quinone is intercalated it cannot be reduced to the semiquinone radical by the reducing enzyme systems.

Adriamycin binds $Fe^{III}$ with great affinity and this complex may be one active form of the drug *in vivo*. The iron mediated cycle of oxygen reduction and free radical generation by adriamycin was described by *J.L. Zweier* (*National Heart, Lung and Blood Institute, Bethesda*). Using the techniques of optical, ESR and Mossbauer spectroscopy, he showed that, while a high spin $Fe^{III}$ complex of adriamycin is rapidly formed, it slowly converts, under anoxic conditions, to high spin $Fe^{II}$, with formation of an oxidized adriamycin free radical. A transient iron-adriamycin radical complex is also observed. The $Fe^{II}$ complex can be converted to the ferric form on exposure to oxygen, with concomitant formation of superoxide anion radicals, which give rise to hydrogen peroxide. In the presence of excess adriamycin, the cycle of oxygen reduction will continue. The oxygen and adriamycin radicals so formed may mediate the therapeutic and toxic effects of the drug. Using $^{13}C$-NMR, the extent of paramagnetic broadening showed that the site of iron binding to adriamycin was in the region of quinone and hydroquinone groups on $C_{11}$ and $C_{12}$, but not those on $C_5$ and $C_6$. The initial site of radical formation in the $Fe^{III}$-adriamycin complex is the side chain of adriamycin. Consequently, in the $Fe^{III}$-daunomycin complex, daunomycin is unable to reduce iron, no free radical from the drug is detected and no oxygen consumption from $O_2^{\cdot -}$ formation is observed.

Mitozantrone is structurally related to the anthracyclines in that it has an anthracycline chromophore and analogous cationic functional groups. In comparing mitozantrone with adriamycin, *L.H. Patterson* (*Department of Pharmaceutical Chemistry, Leicester Polytechnic*) demonstrated that adriamycin gives an ESR signal in rat hepatocytes in which glutathione synthesis is inhibited. This ESR signal was attributed to the adriamycin semiquinone. However, mitozantrone at the same concentrations does not give an ESR signal. Neither drug induced lipid peroxidation but interestingly, mitozantrone is significantly more toxic than adriamycin. It was also suggested that the toxic effects of adriamycin on heart tissues could be brought about by reactions of the drug with oxymyoglobin. It is generally believed that adriamycin semiquinone and superoxide radicals are produced *in vivo* by one-electron reducing enzymes. It was shown, however, that adriamycin reacts directly with oxymyoglobin resulting in the production of superoxide radicals and metmyoglobin. The metmyoglobin undergoes reactions with hydrogen peroxide to produce a peroxidase system. The eventual product from adriamycin in these reactions was shown to be an extremely insoluble precipitate which was not the normal adriamycin aglycone.

Although ESR is a powerful technique for identification of free radicals, the signals obtained on enzymatic reduction of anthracyclines consist of single lines, which do not allow identification, although, from their g-value, they are assigned to semiquinone radicals. *R.P. Mason* (*National Institute of Environmental Health Sciences, Research Triangle Park*) showed that, by addition of the non-aqueous solvents ethanol or dimethyl sulphoxide, it was possible to obtain spectra that were highly resolved. With the aid of computer simulations and of selective deuteration of exchangable protons, it has been possible to obtain positive identifications of the radicals. The radical obtained on enzymic reduction of daunomycin changes with time, becoming more immobilized and the ESR spectrum changing from isotropic to axially symmetric. By addition of non-aqueous solvent, it can be shown that the daunomycin semiquinone changes, with time, to the 7-deoxydaunomycinone semiquinone and it is suggested that this may form aggregates, that are immobilized, on the ESR time scale. Dr. Mason also presented recent results showing the reduction of various naphthoquinones, by glutathione, to the corresponding semiquinones, with concomitant conjugation to glutathione.

α-Naphthol inhibits protein and DNA synthesis and the growth of colonic tumour cells. *In vitro* it is oxidised by mixed function oxidases to 1,2- and 1,4-naphthoquinones and has been shown to be selectively toxic *in vitro* towards certain tumour cells compared to the normal cell lines. *G.M. Cohen* (*School of Pharmacy, University of London*) discussed the many mechanisms of activation and selectivity of α-naphthol and explained that naphthol can form sulphonated ester conjugates or glucuronide adducts. In certain tumours the major product is the glucuronic acid conjugate and this is one difference between the tumour cell compared to the normal cell. α-Naphthol also leads to a depletion in intracellular glutathione and dicoumarol increases this depletion and the toxicity. This implies that the one-electron reduction processes are more damaging than the two-electron processes. Purified 1,2- and 1,4-naphthoquinones are also toxic and are similarly affected by dicoumarol. α-Naphthol has also been shown to be a substrate for peroxidase and inhibits ribonucleotide reductase, possibly by scavenging the tyrosine radical at the active site.

Continuing the theme of naphthoquinones, *S. Orrenius* (*Karolinska Institute, Stockholm*) discussed the mechanisms of menadione (2-methyl-1,4-naphthoquinone)-induced damage in various intact cell systems and in particular, the rat hepatocyte. The marker for toxicity is the formation of characteristic blebs on the cell surface which is an early morphological sign of cell injury. The extent of blebbing can be modified by dicoumarol or by glutathione inhibitors. The quinone can be reduced in hepatocytes by one- or two-electron processes, the latter is inhibited by dicoumarol, or can add directly to GSH and -SH groups in proteins. Both the reduction and addition processes lead to a net GSH and protein -SH depletion. During these processes there is a mobilisation and loss of calcium from the mitochondrial and extramitochondrial compartments and an increase in cytosolic calcium. Among its many effects, calcium influences actin binding capacity which may be partly responsible for the onset of blebbing. The discussion at the end of this talk was extensive and it was generally accepted that this type of mechanism could also occur for some antitumour drugs.

*J.R. Brown* (*Department of Pharmaceutical Chemistry, Sunderland Polytechnic*) posed a number of questions regarding the initial steps of bioactivation of anthraquinones, the first of which was whether the oxidation of the hydroquinone ring of adriamycin and daunomycin could be significant as well as the more widely studied reduction. With respect to the latter, could two-electron reduction be used to avoid oxygen-linked problems associated with the formation of the semiquinone? He wondered what information about the reduction could be obtained from the biological data. Were DNA binding or redox cycling of greater significance? Computer graphics and kinetic studies had been made of the binding to DNA of a number of substituted anthraquinones. This property depends markedly on substitution. All of the compounds could be reduced by mouse liver microsomes. They varied in the effectiveness with which they could generate $O_2^{\cdot -}$. This was not due to differences in one-electron reduction potential but because of differing abilities to act as substrate for the enzyme.

The chemistry of semiquinones whether formed via one-electron oxidation or one-electron reduction, and other intermediates, can be investigated in an effective way by pulse radiolysis. *A.J. Swallow* (*Paterson Institute for Cancer Research, Manchester*) showed how the species can be formed in aqueous solutions, and how optical absorption spectra, values of $pK_a$, thermodynamic reduction potential and reaction rate constants can be obtained. Naphthazarin, a model compound for adriamycin, has a reduced semiquinone which is extraordinarily stable. The reduced semiquinones of adriamycin and daunomycin are even more stable with respect to dismutation, but decay by intramolecular processes within about a second. The semiquinone of mitomycin C readily dismutes. The hydroquinone form undergoes two pH-dependent principal reactions over seconds. Reduction products as identified by HPLC are the same whether reduction is effected pulse radiolytically or enzymically.

AZQ (diaziquone,2,5-diaziridinyl-3,6-bis(carbethoxyamino)-1,4-benzoquinone) is currently undergoing clinical trials. *J. Butler* (*Paterson Institute for Cancer Research, Manchester*) reported on the consequences of one-electron reduction of this and a number of related compounds as studied by pulse radiolysis and HPLC. AZQ crosslinks DNA, induces strand breaks and produces free radicals when reduced by a number of mild reductants. Its semiquinone dismutes hundreds of times more slowly than some other semiquinones. The resulting hydroquinone undergoes aziridine ring opening over a period of minutes. The rate of the reaction depends on pH. In the presence of oxygen the hydroquinones thus formed react to give ring opened quinones. He suggested that the reaction of the hydroquinone with oxygen is not a two-electron process but rather produces, in the first instance, a semiquinone radical and $O_2^{\cdot -}$. The semiquinone then reacts with oxygen to give more $O_2^{\cdot -}$. The superoxide radicals can dismute to form $H_2O_2$. Such one-electron steps could be common if not universal intermediate stages in oxidation by oxygen.

Mitomycin C and porphyromycin are more toxic towards hypoxic cells than fully oxygenated cells. *S.R. Keyes* (*Comprehensive Cancer Center, Yale University*) discussed the various mechanisms of toxicity and reported on a preliminary clinical trial on the use of these drugs and dicoumarol in combination with radiotherapy. Dicoumarol significantly increased the toxicity of mitomycin C and radiation towards tumour cells but it is not in itself a radiosensitiser nor toxic. In hypoxia the NADPH-cytochrome c reductase activity is at least partly responsible for the activation of mitomycin C to toxic products. Dicoumarol, a supposedly specific inhibitor of the DT diaphorase system, results in an increase in the formation of reactive electrophiles for mitomycin C under hypoxic conditions implying that it is inhibiting an enzyme that would normally metabolize the drug to non-active products. Dicoumarol also eliminates the DNA cross links produced by mitomycin C suggesting that this type of cross link is not solely responsible for the toxicity of the drug. Evidence was presented that suggests protein-DNA cross links may be more important to the toxicity in hypoxic conditions. Probably one of the most controversial points of this presentation was that dicoumarol is not necessarily an inhibitor of the DT diaphorase system. L1210 cells have high NADPH-cytochrome c reductase activity but no measurable DT diaphorase activity. However, the increase in toxicity produced by dicoumarol is still present in hypoxic L1210 cells. This suggests that dicoumarol inhibits at least one other enzyme besides the diaphorase system.

*M. Tomasz* (*Hunter College, City University of New York*) described the first reported isolation and structure determination of the major covalent adducts formed on reaction of DNA with chemically or enzymatically activated mitomycin C. Activation of mitomycin C in the presence of calf thymus DNA using NADPH-cytochrome c reductase, xanthine oxidase or $H_2/PtO_2$ was found to result in covalent complex formation. Digestion of the complex followed by HPLC yielded $N^2$-(2″β,7″diaminomitosen-1″α-yl)2′deoxygaunosine (I) as the major DNA alkylation product together with two minor monoadducts identified as the 1″β stereoisomer of I and 10′-decarbamoyl-I, proof of these structures being gained by 1H NMR, MS, FTIR and CD. The same results were obtained with poly (dG-dC).poly(dG-dC), although in this case an additional minor adduct peak was found due to a crosslinking adduct. The latter resulted from the addition of one mitomycin to two guanosines, the second guanosine being attached at the 10′ position. This biadduct was also isolated from rats injected with mitomycin C. Space-filling molecular model building indicates a snug fit of the guanine $N^2$-linked drug monoadduct inside the minor groove of B-DNA with no appreciable distortion of the DNA structure, and also that the biadduct is even less disturbing to DNA than the monoadduct.

Beginning the final morning, *J.M. Bruce* (*Department of Chemistry, University of Manchester*) presented an overview of quinone activity informed by his own knowledge as an organic chemist, commenting upon mechanistic aspects of some of the earlier presentations. Quinones, anti-aromatic diketone systems which, when reduced by single electrons, give the corresponding aromatic semiquinone radicals, have inherent reactivity and on bioactivation can lead to new species of differing reactivities. Under hypoxic conditions, e.g. in solid tumours, quinones can be reduced to semiquinones. The ability of quinones to accept electrons is reflected in their half-wave reduction potentials which can be raised by substituents which stabilise the semiquinone, e.g. CN and Cl, and lowered by others which destabilise $Q^{\cdot -}$, e.g. $CH_3$ and $CH_3O$. If some oxygen is present, semiquinones may react to give superoxide which can ultimately lead to oxygen toxicity via $H_2O_2$ and $OH^{\cdot}$. Quinones can also decay to give other active species such as quinone methides which are likewise reactive towards nucleophiles. As a supporter of quinone methide intervention in anticancer drug activity, Dr. Bruce suggested that at least one of the covalent bonds resulting in the adducts described by Dr Tomasz result from an interaction of a quinone methide derived from mitomycin C.

The subject of polarographic half-wave potentials was further developed by *J.M. Moiroux* (*Ecole Normale Superieure de l'Enseignement Technique, Cachan*) who described a spectroelectrochemical study of the 1- and 2-electron reduction of a group of substituted anthraquinones in aprotic medium (DMF), the ultimate aim being to see if there is any correlation between anthracycline reducibility and cytotoxicity and/or cardiotoxicity. Only the anthraquinone derivatives without hydroxy substituents had reduction potentials more negative than the $O_2/O_2^{\cdot -}$ couple, yet for the hydroxyanthraquinones the radical anions were still oxidised by oxygen, the electron transfer being accompanied by a proton transfer between the OH group and $O_2^{\cdot -}$, yielding $HO_2^{\cdot}$ and the conjugate base of the original anthraquinone. In DMF the semiquinone of daunomycin was found to be stable, glycoside elimination and formation of the basic form of the quinone methide only occurring after addition of the second electron. Kinetic studies by reverse pulse polarography showed that the glycoside elimination rate varied on introducing protons into the medium and also with the structure of the sugar moiety.

The conference concluded with a description by *H. Berg* (*Zentralinstitüt fur Mikrobiologie und Experimentelle Therapie, Jena*) of a broad, mainly physicochemical, study of a series of eight anthracyclines: aclacinomycin A, adriamycin, daunomycin, $\beta$-rhodomycin-II, $\beta$-rhodomycin-I, carminomycin, iremycin and 5-imino daunomycin. The anthracycline properties investigated included dimerisation, equilibrium and kinetic constants for their complexes with DNA, nucleoproteins and oligonucleotides, redox reactions, deglycosidation and peroxide formation. A close correlation was found between the anthracycline-DNA binding constant and drug antimicrobial activity against *Bacillus subtilis* ATCC 6633. The kinetics of daunomycin-DNA association followed by the temperature jump technique fitted two exponentials. Aclacinomycin was found to have the highest affinity for DNA, the largest binding area (4 base pairs), to be a more efficient inhibitor of DNA and RNA synthesis than adriamycin or daunomycin, and to have the most positive reduction potential, recorded electrochemically. correlating with low cardiotoxicity. A general conclusion from all these studies, consistent with the results presented in several other contributions at this symposium, was that the cytotoxic effects of anthracyclines are likely to operate via several different molecular mechanisms.

In conclusion, the contents of the symposium, chosen to be on a limited topic, amply demonstrate the wide range of approaches currently being enthusiastically applied to studies of the bioactivation of quinone anti-tumour agents. The high quality of the talks, and the lively discussions which followed each, encourage the feeling that, during the next few years, the fundamentals of this aspect of cancer chemotherapy should become established, leading to the production of valuable new drugs.

The symposium was supported by the Christie Hospital General Research Endowment Fund. The Cancer Research Campaign is thanked for a grant permitting the attendance of three younger scientists as observers.